# Observation of near-inertial waves in the wake of four typhoons in the northern South China Sea

**DOI:** 10.1038/s41598-023-29377-3

**Published:** 2023-02-23

**Authors:** Qinglong Gong, Qixiang Wang, Liang Chen, Yina Diao, Xuejun Xiong, Jilin Sun, Xianqing Lv

**Affiliations:** 1 Frontier Science Center for Deep Ocean Multispheres and Earth System (FDOMES) and Physical Oceanography Laboratory, Ocean University of China, Qingdao, 266100 China; 2 First Institute of Oceanography, Ministry of Natural Resources, Qingdao, 266061 China; 3 Founctional Laboratory for Regional Oceanography and Numerical Modeling, Pilot National Laboratory for Marine Science and Technology (Qingdao), Qingdao, 266237 China; 4 Laboratory for Regional Oceanography and Numerical Modeling, Pilot National Laboratory for Marine Science and Technology (Qingdao), Qingdao, 266237 China; 5 Qingdao National Laboratory for Marine Science and Technology, Qingdao, 266200 China

**Keywords:** Climate sciences, Ocean sciences

## Abstract

Based on the velocity and temperature data recorded by two acoustic Doppler current profilers (ADCPs) at a mooring system deployed in the northern South China Sea (SCS), this study investigates the characteristics of near-inertial waves (NIWs) induced by typhoons Bebinca, Barijat, Mangkhut and Yutu in 2018. For the dynamical response, besides the motion of near inertial frequency induced by typhoons, the motion of 2 f ([1.80–2.20] *f*,* f* is the local inertial frequency) and *f* D_1_ (a harmonic wave with a frequency equal to the sum of frequencies of NIWs and diurnal tides) frequency will also increase. For near-inertial motions, the maximum near-inertial kinetic energy (NIKE) is confined to depths above 150 m. For stronger (weaker) wind forcing, the longer (shorter) the response time of the ocean to the atmospheric forcing is, and the shorter (longer) the response time is required in relaxation stage. There are upward and downward propagating energies after the passage of typhoons, and the upward propagating energy mainly occur in the stage of the geostrophic balance adjustment. The current structure suggests that the NIWs in the vertical direction are two antisymmetric rotary vortices in a near-inertial period, which is similar to the structure of the Langmuir circulation. Besides, the horizontal near-inertial currents (NICs) are much stronger than the vertical NICs, and the stronger the NIWs are, the stronger the horizontal NICs relative to the vertical NICs are. For the temperature response, the temperature variation reflects a clear stratified vertical structure. In the forcing stage, the upper layer becomes colder, the lower layer becomes warmer, and the thickness and intensity of the thermocline decrease. In the relaxation stage, the upper layer warms and the lower layer cools, and the thickness and intensity of thermocline increase.

## Introduction

Near-inertial waves (NIWs) are common internal waves in the ocean. Its most obvious feature is that it appears as a peak near the local inertial frequency *f* in the power spectrum, which is rising well above the continuous internal wave spectrum. If factors such as background vorticity are not considered, the frequency of NIWs is usually slightly larger than the local inertial frequency^1^. The horizontal scale of NIWs is usually hundreds of kilometers^2^, and the vertical scale range from a few meters to hundreds of meters^3^, with circular polarization velocities (clockwise in the northern hemisphere)^1^.

In the equations of motion, the excitation of near-inertial motion is the result of the resonance of the local inertial frequency, so in essence, any force can excite NIWs. In the ocean, the main generation mechanisms of NIWs include wind forcing at the sea surface^4–6^, nonlinear wave-wave interactions such as parametric subharmonic instability^7,8^, the formation of lee waves by the geostrophic currents over the seafloor topography^9,10^, and the geostrophic adjustments caused by the frontogenesis and radiation by time-dependent instabilities of low-frequency flow lead to spontaneous generation^11–14^, in which the mechanism of wind generated NIWs is considered to be the most effective mechanism, especially when typhoons pass by^1^. Hou et al. analyzed NIWs events generated by different mechanisms^15^, and considered that the NIWs generated by typhoons were the most regular. Since NIWs account for most of the energy in the internal wave spectrum^16,17^, and have strong vertical shear which dominated by small vertical scale^1,18–22^, NIWs with a small vertical scale of a few meters play a role in the mixing of oceans^23–25^.

When a typhoon passes over the ocean, the momentum and heat exchange at the air-sea interface will cause dynamic and thermodynamic responses in the upper ocean. The dynamic response refers to the seawater flows and ocean fluctuations excited by the typhoon in the upper ocean, and the thermodynamic response refers to the mixing of cold and warm water masses at different depths^26–30^. For a moving typhoon, the response of the upper ocean can basically be divided into two stages: forcing stage and relaxation stage^27,30,31^. The seawater motion excited by typhoon is mainly manifested as a significant enhancement of near-inertial motion^32^, especially in the upper layer, which can persist 5–20 inertial periods before decaying^1,4^. For example, the near-inertial currents (NICs) excited by typhoon in the South China Sea (SCS) can reach 0.5 m/s^33^. The near-inertial kinetic energy (NIKE) corresponding to the NICs is also enhanced, reaches the maximum in the mixed layer, and even exceeds three times the internal tidal energy^34,35^. There is vertical propagation of NIWs excited by typhoons^36^, the vectors rotate clockwise in depth indicating a downward energy propagation, and NIKE usually decreases rapidly after passing through the thermocline^37,38^. NIKE excited by typhoons is already weak when it propagates to a depth below 500 m^39^. In addition to vertical propagation, NIWs also have horizontal propagation due to *β* effect, mainly including equatorial and polar propagation. NIWs with frequencies less than *f* can propagate equatorward until their frequencies become 2 f and dissipate due to parametric subharmonic instability, while NIWs with frequencies greater than *f* can propagate poleward until they reach the turning latitude^1,16^. The horizontal trajectories of NIWs are clockwise rotating circles following the cyclones^4^. The NICs excited by typhoons are generally related to the intensity of typhoons, including the maximum wind speed of typhoons, the radius of maximum wind (distance from the center of a cyclone to its strongest wind band) and the moving velocity of typhoons, and there is a positive correlation between them^40^. Besides, NICs are also related to the position of typhoons, and the ocean usually has a stronger near-inertial response on the right side of the typhoon track^27,41^. The intensity of NICs generated by typhoons in different sea areas is also different. In the SCS, the NICs generated by typhoons^32^ are smaller than those in the Atlantic region^42,43^ and the Bay of Bengal^44^, and the maximum NICs in the latter two regions both exceeds 1.2 m/s.

As the largest marginal sea in the Northwest Pacific, the SCS is vulnerable to typhoons, with an average of 10.2 typhoons occurring or passing each year^45^, so it is an ideal area to study the NIWs generated by typhoons. We deploy two moored acoustic Doppler current profilers (ADCPs) in the SCS to measure current profiles covering the water depth, and obtain 131 days continuous time series. A total of 4 typhoon events with different intensities are captured during the observation period. In this study, the features and correlations of NIWs generated by 4 typhoons are analyzed, and the reasons for the differences are tried to be explored. This paper is organized as follows. Results show and analyze the observation of NIWs. Discussion analyzes kinetic energy, thermodynamic response and current structure of ISWs. Summary gives study conclusions.

## Data and methods

The data used in this paper include the European Center for Medium-Range Weather Forecasts (ECMWF) Reanalysis 5th Generation (ERA5) dataset provided by the ECMWF, the best track dataset of tropical cyclone provided by the China Typhoon Network, and observation data recorded by ADCPs deployed in the northern SCS.

### ERA5 dataset

**Figure 1:**
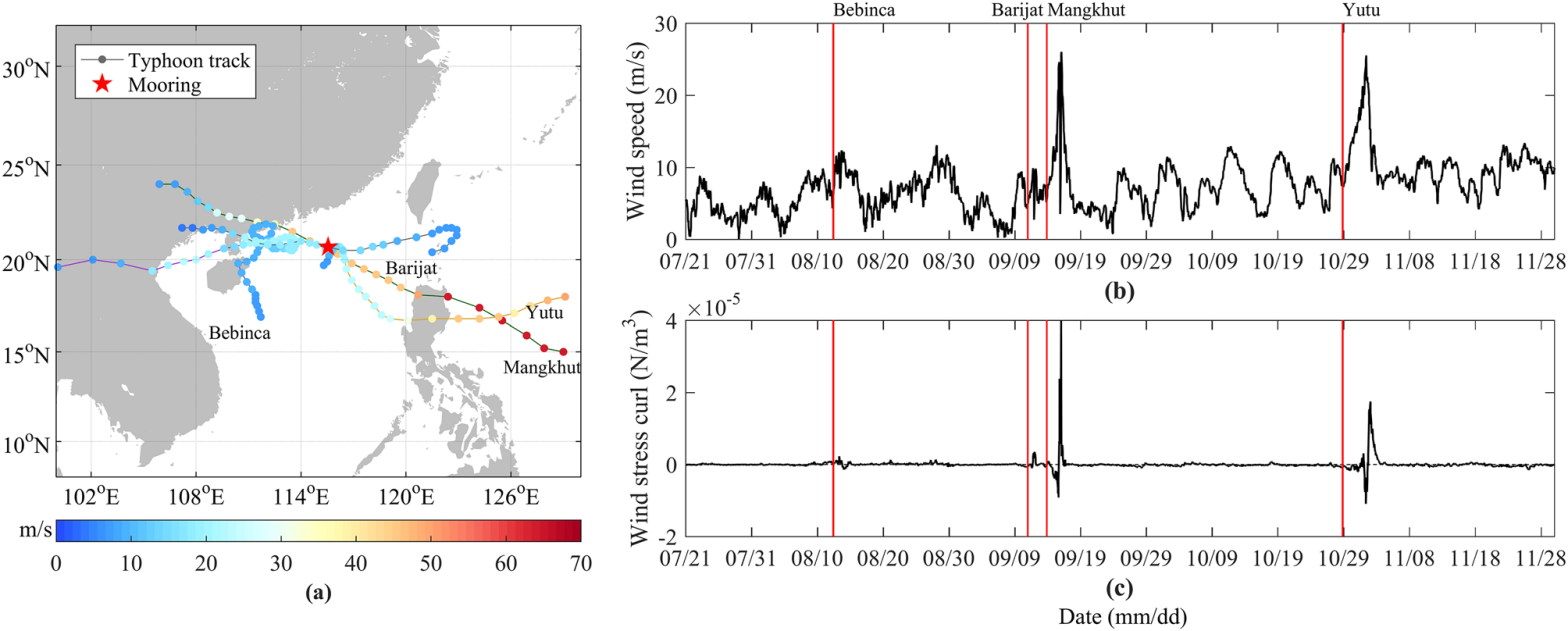
Map of the mooring near the Dongsha Islands in the SCS (**a**) and the time series of 10 m wind speed (**b**) and wind stress curl (**c**) over the sea surface at the mooring extracted from ERA5 data. The red pentagram denotes the mooring position. The solid lines with different colors denote the tracks of the four typhoons. The dots with different colors denote the maximum wind speed at the center of typhoons, and the unit of wind speed is m/s. The red vertical lines represent the time when typhoons passed. Figures are plotted using MATLAB R2016b (http://www.mathworks.com/).

Hourly 10 m wind speed derived from ERA5 dataset is used to calculate the wind stress and wind stress curl at 10 m over the sea surface, and the time range is from July 1, 2018 to November 30, 2018. The time resolution is 1 h, and the spatial resolution is 0.25 × 0.25°. Dataset can be found at https://www.ecmwf.int/en/forecasts/datasets/browse-reanalysis-datasets. Figure 1b,c shows the wind speed and wind stress curl at the mooring during the observation period. The passing time of typhoons Mangkhut and Yutu can be clearly known from the change of wind speed (Fig. 1b). The wind speed excited by typhoons Bebinca and Barijat at the mooring is weak, so the time series of the wind stress curl is given (Fig. 1c). From the wind stress curl, the specific stage of each typhoon passing through the mooring can be clearly seen, and the start time of each typhoon affecting the mooring is determined in combination with the wind speed (Table 1).

**Table 1:** Typhoon information affecting mooring during observation period.

Typhoon	Before the passage	After the passage
Bebinca	08/07 08:00–08/12 11:00	08/12 11:00–08/30 12:00
Barijat	09/01 00:00–09/11 00:00	09/11 00:00–10/07 12:00
Mangkhut	09/01 00:00–09/13 21:00	09/13 21:00–10/07 12:00
Yutu	10/20 05:00–10/28 20:00	10/28 20:00–11/20 06:00

### Typhoon dataset

The typhoon best track dataset is provided by the China Typhoon Network (tcdata.typhoon.org.cn), including the typhoons’ central longitude and latitude, central minimum pressure, 2-min averaged maximum wind speed near center, with a temporal resolution of 6 h and a spatial resolution of 0.1°^46,47^. During the observation period, a total of 4 typhoons affect the mooring, namely Bebinca, Mangkhut, Barijat and Yutu. The specific information is shown in Table 1, and the typhoon tracks and the mooring position are shown in Fig. 1a.

### In situ observation data

During the period from 21 July to 20 December 2018, a mooring is deployed on the continental slope area (115.55ºE, 20.68ºN) on the west side of the Dongsha Islands in the northern SCS to measure temperature, salinity and current velocity. The water depth of the observation station is 662 m, and the current velocity is measured by a downward-looking 300 kHz ADCP and an upward-looking 75 kHz ADCP at the depth of 580 m with a vertical resolution of 8 m and time interval of 3 min. The temperature and depth are measured by conductivity-temperature-depth (CTD) at every 50 m and by temperature loggers at every 10 m, and the sampling interval of CTDs and temperature loggers both are 30 s.

### Methodology

The relationship between wind stress and wind speed is:

1 $$\mathop{\tau }\limits^{\rightharpoonup} = \rho_{a} C_{d} U_{10} {\mathop{u}\limits^{\rightharpoonup}}_{10} ,$$

where ${\rho }_{\text{a}}$ is the density of air, ${\text{C}}_{\text{d}}$ is the drag coefficient, which is calculated according to the methods of Oey et al.^48^, ${\text{U}}_{10}$ and $\mathop{u}\limits^{\rightharpoonup} _{10}$ are the wind speed magnitude and wind speed vector at 10 m above the sea surface, respectively.

The near-inertial current velocity is extracted from the observed current velocity using a bandpass filter. The cut-off frequency of the near-inertial current velocity is [0.80–1.20] *f* (~ 0.57–0.85 cycles per day (cpd) at mooring station), where *f* is the local inertial frequency. In addition, 2 f ([1.80–2.20] *f*), *O*_1_, *K*_1_, *f O*_1_, *f K*_1_, *M*_2_ and *S*_2_ are also used in this study, where* O*_1_ (~ 0.93 cpd) and *K*_1_ (~ 1.00 cpd) are diurnal internal tides, *M*_2_ (~ 1.93 cpd) and *S*_2_ (~ 2.00 cpd) are semidiurnal internal tides, *f O*_1_ (*f* + *O*_1_, ~ 1.64 cpd) and *f K*_1_ (*f* + *K*_1_, ~ 1.71 cpd) are harmonic waves with a frequency equal to the sum of the frequencies of NIWs and diurnal internal tides. The frequency bands from *O*_1_ to *K*_1_, from *f O*_1_ to *f K*_1_, and from *M*_2_ to *S*_2_ are defined as *D*_1_, *f D*_1_ and *D*_2_ bands, respectively.

Power spectrum can transform the time domain signal into the frequency domain signal, so as to extract the periodic signal in the motion. Power spectrum estimation in this study is calculated according to the method proposed by Welch^49^, which includes the following steps: (a) divide the observed time series into overlapping *K* segments; (b) calculate the periodogram after adopting a hamming window to each time series (time-domain windowing represents the point multiplication of the time domain signal and window function); (c) the power spectrum estimation is calculated by averaging the periodogram.

The velocity rotation spectrum is used to analyze the rotation characteristics of the current velocity. The time series $u(t)+ iv(t)$ of the horizontal current velocity can be decomposed into a series of different frequencies ω^50^:

2 $$u\left( \omega \right) + iv\left( \omega \right) = \frac{1}{T}\int_{0}^{T} {\left[ {u\left( t \right) + iv\left( t \right)} \right]e^{ - i\omega t} dt} ,$$

where, *T* is the duration of the record. The current velocity $u(\omega) + iv(\omega)$ is decomposed into the superposition of clockwise rotation component ${u}_{-}(\omega )$ and counterclockwise rotation component ${u}_{+}(\omega )$:

3 $$u\left( \omega \right) + iv\left( \omega \right) = u_{ + } \left( \omega \right)e^{i\omega t} + u_{ - } \left( \omega \right)e^{ - i\omega t} .$$

The spectra of the clockwise and counterclockwise rotation components are defined as follows:

4 $$\left\{ {\begin{array}{*{20}c} {S_{ - } = \frac{1}{2}\left\langle {u_{ - }^{*} u_{ - } } \right\rangle } \\ {S_{ + } = \frac{1}{2}\left\langle {u_{ + }^{*} u_{ + } } \right\rangle } \\ \end{array} } \right.,$$

where, ${S}_{-}$ (${S}_{+}$) is the clockwise (counterclockwise) rotary spectrum, ${u}_{-}$ (${u}_{+}$) is the clockwise (counterclockwise) rotary component, the angular brackets denote the average over all the pieces), and the stars denote the complex conjugate.

Wave number rotation spectrum is often used to reveal the direction of energy propagation in NIWs. The vertical distribution of horizontal velocity $u(t)+iv(t)$ can be written as the component superposition of different vertical wave numbers *m*:

5 $$u(m) + iv(m) = \frac{1}{H}\int_{ - H}^{0} {[u(z) + iv(z)]} e^{ - imz} dz,$$

where, *H* is the water depth. The current velocity $u(m)+iv(m)$ is decomposed into the sum of depth-dependent clockwise rotation component ${u}_{-}(m)$ and depth-dependent counterclockwise rotation component ${u}_{+}(m)$:

6 $$u(m) + iv(m) = u_{ + } (m)e^{imz} + u_{ - } (m)e^{ - imz} .$$

In this case, the clockwise and counterclockwise rotation component spectra are defined as follows^51^:

7 $$\left\{ {\begin{array}{*{20}c} {C_{m} = \frac{1}{2}\left\langle {u_{ - }^{*} u_{ - } } \right\rangle } \\ {A_{m} = \frac{1}{2}\left\langle {u_{ + }^{*} u_{ + } } \right\rangle } \\ \end{array} } \right.,$$

where, ${C}_{m}$ (${A}_{m}$) is the clockwise (counterclockwise) rotary spectrum, ${u}_{-}$ (${u}_{+}$) is the clockwise (counterclockwise) rotary component, the angular brackets denote the average over all the pieces), and the stars denote the complex conjugate. The energy propagation direction is indicated by ${C}_{m}-{A}_{m}$, when ${C}_{m}-{A}_{m}$ is positive (negative), the energy propagates downward (upward).

The KE at different frequencies is calculated by the following formula^40^:

8 $$KE_{f} = \frac{1}{2}\rho \left( {u_{f}^{2} + v_{f}^{2} } \right),$$

where, ${\text{u}}_{f}$ and ${\text{v}}_{f}$ are the zonal and meridional velocities at different frequencies, respectively. $\rho$ is the seawater density.

We use the gradient method to determine the depth of the thermocline, consider the depth where the temperature vertical gradient exceeds 0.05 °C/m as the thermocline, and choose the depth with the largest temperature vertical gradient as the depth of the strongest thermocline. Due to the lack of upper layer observation temperature data, this paper only considers the region between the strongest thermocline and the lower boundary of the thermocline. In this paper, the upper boundary layer of the thermocline refers to the depth of the strongest thermocline, and defines the average thermocline intensity as

9 $$I = \,\left( {t_{l} - t_{m} } \right)/\left( {d_{m} - d_{l} } \right),$$

where, $I$ is the average thermocline intensity, ${t}_{l}$ and ${t}_{m}$ are the temperature at the lower boundary of the thermocline and at the depth of the strongest thermocline, respectively. ${d}_{l}$ and ${d}_{m}$ are the depth of the lower boundary of the thermocline and the depth of the strongest thermocline, respectively.

## Results

### Observed current velocity

**Figure 2:**
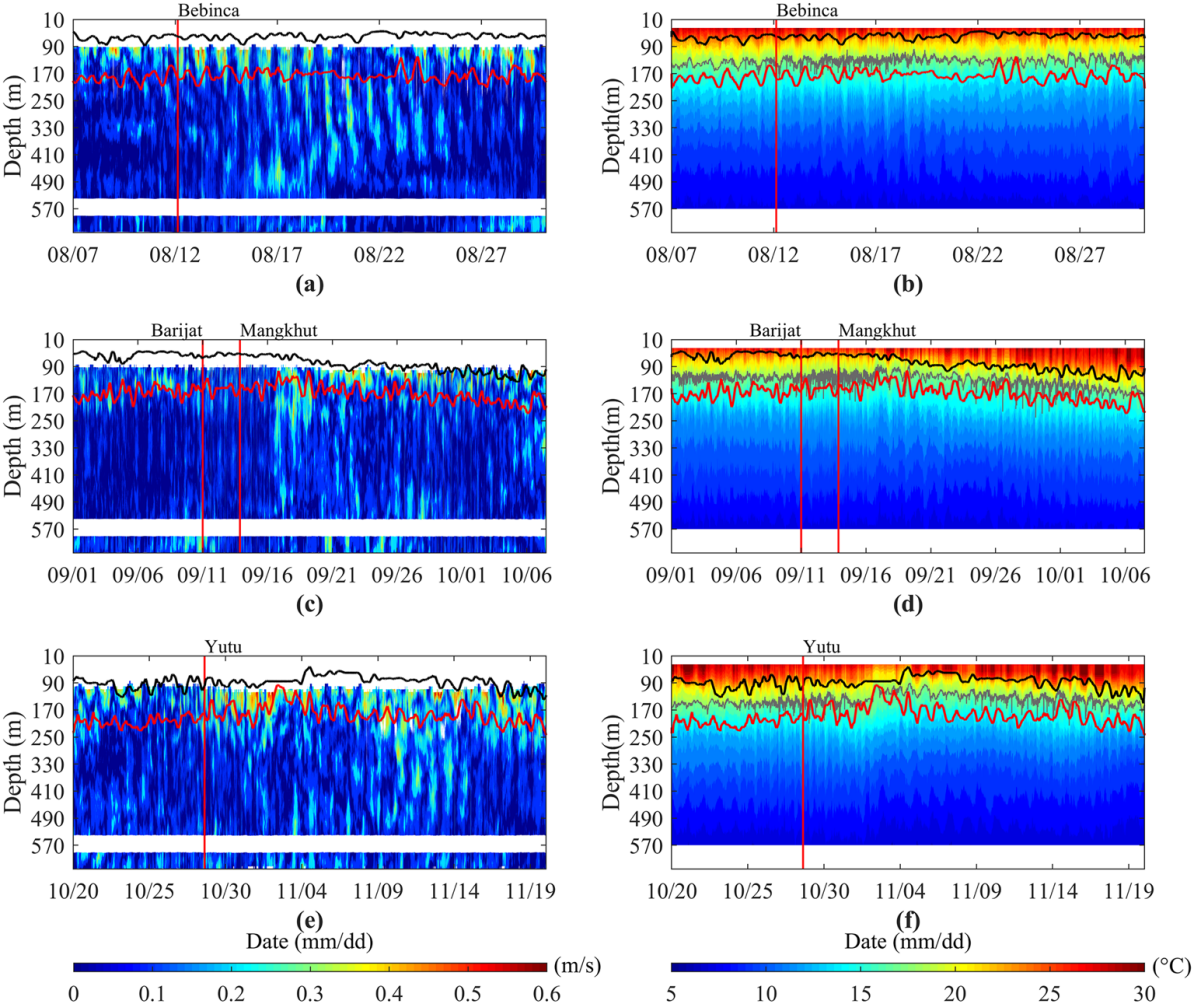
Time series of the current velocity (**a,c,e**) and temperature (**b,d,f**) during the passage of typhoons Bebinca (**a,b**), Barijat and Mangkhut (**c,d**), and Yutu (**e,f**) versus depth, respectively. The x-axis is in the format of month/day. The red vertical lines are same as Fig. 1. The red horizontal lines are the lower boundary of the thermocline. The gray horizontal lines are 18 °C isotherm, and the black horizontal lines are the depth of the strongest thermocline.

The time series of observed current velocity and temperature versus depth, the depth of the strongest thermocline and the lower boundary of the thermocline during the passage of typhoons are shown in Fig. 2, the upper boundary of the thermocline is not given here due to the absence of temperature observations in the upper layer. It can be seen that the total observed current velocity increase significantly for typhoons Bebinca, Mangkhut and Yutu, which is more obvious in the subsurface layer. After the passage of typhoon Bebinca, the maximum observed current velocity in the subsurface layer increase from 0.76 m/s to 1.02 m/s. After the passage of typhoon Mangkhut, the maximum observed current velocity in the subsurface increase from 1.02 m/s to 1.40 m/s. After the passage of typhoon Yutu, the maximum observed current velocity in the subsurface increase from 0.77 m/s to 1.05 m/s. However, for typhoon Barijat, the observed current velocity is not strong, and after it passed, the maximum observed current velocity in the subsurface increase from 0.66 m/s to 0.71 m/s.

During the passage of the typhoons, the same characteristics of change in subsurface temperature can be seen, first becoming colder and then warmer. The temperature change is manifested as an upward and downward movement of the isothermal surface. For the thermocline, the lower boundary of the thermocline shows a trend of becoming shallower first and then deeper during the passage of the typhoons, especially in the case of typhoons Barijat, Mangkhut and Yutu. Before the passage of typhoons, the average depth of the lower boundary of the thermocline is 184.88 m, 162.52 m and 208.54 m respectively. After the passage of typhoons, the shallowest lower boundary depth of the thermocline reaches 123.25 m, 102.34 m and 96.52 m respectively. For the depth of the strongest thermocline, after the passage of typhoons Bebinca and Yutu, the depth of the strongest thermocline is shallower first and then deeper. In the whole stage after the passage of typhoon Barijat and Mangkhut, the depth of the strongest thermocline presents a process of continuous deepening. In addition, after the passage of typhoons, each strong current velocity corresponds to the shallower depth of the lower boundary of the thermocline, which indicates that the strong current caused by the typhoon will change the structure of the thermocline and thus affect the temperature distribution.

### Spectral analysis

**Figure 3:**
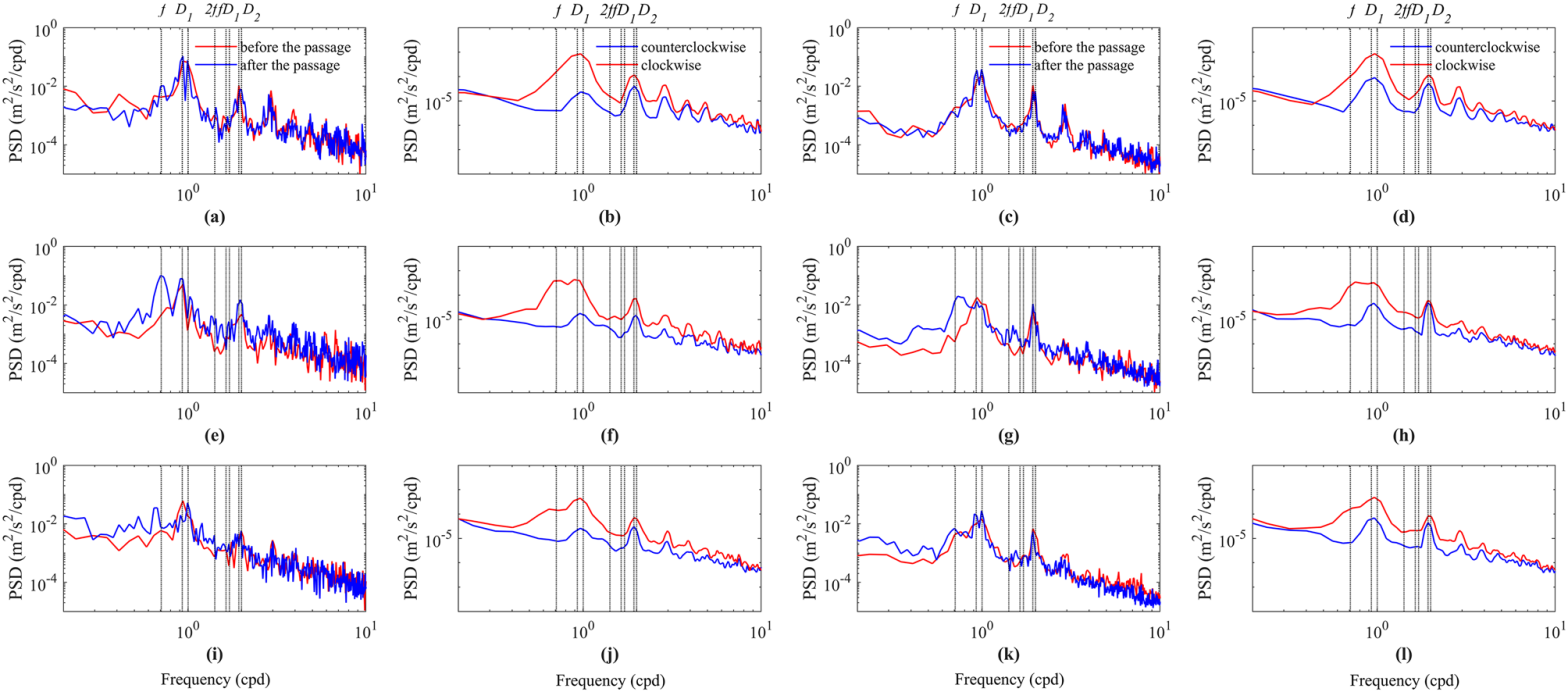
Depth-averaged power spectra and rotary spectra of observed current velocity in the thermocline (**a,b,e,f,i,j**) and subthermocline (**c,d,g,h,k,l**) the lower boundary of thermocline during the passage of typhoons Bebinca (**a–d**), Barijat and Mangkhut (**e–h**), and Yutu (**i–l**), respectively. Here and subsequently, the eight black vertical lines from the left to the right in each subfigure denote where the frequencies of depth-averaged near-inertial oscillation in the whole water column, *O*_1_, *K*_1_, 2 f, *f O*_1_, *f K*_1_, *M*_2_ and *S*_2_ tidal constituents are situated, respectively. The frequency bands from *O*_1_ to *K*_1_, from *f O*_1_ to *fK*_1_, and from *M*_2_ to *S*_2_ are defined as *D*_1_, *f D*_1_ and *D*_2_ bands, respectively.

Figure 3a,e,i are the depth-averaged power spectra of the observed current velocity in the thermocline (above the lower boundary of thermocline) before and after the passage of typhoons. The results show that the thermocline of mooring position is dominated by diurnal internal tides and semidiurnal internal tides before the passage of typhoons, and the diurnal internal tides have a greater spectral energy (the range of power spectral density (PSD) of peaks are about 0.049–0.073 m^2^/s^2^/cpd) than semidiurnal internal tides (the range of PSD of peaks are about 0.004–0.010 m^2^/s^2^/cpd). After the passage of typhoons, the NICs increase, and the intensity of its increase is related to many factors such as the effect of the typhoon on the mooring position. After the passage of typhoons Bebinca and Yutu, the near-inertial oscillations intensify, but do not exceed spectral energy of the diurnal tides, while after the passage of typhoons Barijat and Mangkhut, the near-inertial oscillations increase significantly and exceed spectral energy of the diurnal tides.

Figure 3c,g,k are the depth-averaged power spectra of the observed current velocity in the subthermocline (below the lower boundary of thermocline) before and after the passage of typhoons. The results show that the subthermocline of mooring position is also dominated by diurnal internal tides and semidiurnal internal tides before the passage of typhoons, and the spectral energy of diurnal internal tides (the range of PSD of peaks are about 0.015–0.023 m^2^/s^2^/cpd) are not significantly different from that of semidiurnal internal tides (the range of PSD of peaks are about 0.007–0.011 m^2^/s^2^/cpd). It is found that the spectral energy of the semidiurnal internal tides in the thermocline is not much different from that of the subthermocline, while the spectral energy of the diurnal internal tides in the thermocline is about three times that of the subthermocline. After the passage of typhoon Bebinca, the NICs do not increase, while the NICs increase significantly after the passage of typhoons Yutu, especially Barijat and Mangkhut. This indicates that typhoons Barijat, Mangkhut and Yutu induce NIWs and propagate to deeper depths of the ocean, while typhoon Bebinca also induces NIWs, but the downward propagation is not strong, which is well studied by Gao et al.^52^.

The rotary spectra of observed current velocity in the whole observational depth during the passage of typhoon is calculated in Fig. 3b,f,j,d,h,l. The results show that all cases exhibit similar features, the spectra have obvious peaks in the frequency bands of diurnal and semidiurnal internal tides, and all of them are dominated by clockwise components. In the near-inertial frequency band, the peaks caused by typhoons Barijat and Mangkhut are more obvious in Fig. 3f,h, while the peaks caused by the other two typhoons are not obvious. At the near-inertial frequency, the NICs are dominated by clockwise components, indicating that the NIKE propagated downward^51,53^.

Note that the near-inertial oscillations revealed in the measurements are not necessarily generated locally, may have propagated from different latitudes within the ocean. For example, in Fig. 3i, the near-inertial frequency is smaller than the local near-inertial frequency after the passage of typhoon Yutu, showing a red-shift phenomenon, which may be related to the propagation from a low-latitude ocean or be related to the doppler shift and shear flow modulation^2,54^. Moreover, after the passage of typhoons, there are obvious power spectrum peaks near 2 f and *f D*_1_, which may be related to the wave-current interaction and will be discussed further in the following section.

### Typhoon-induced NIWs

**Figure 4:**
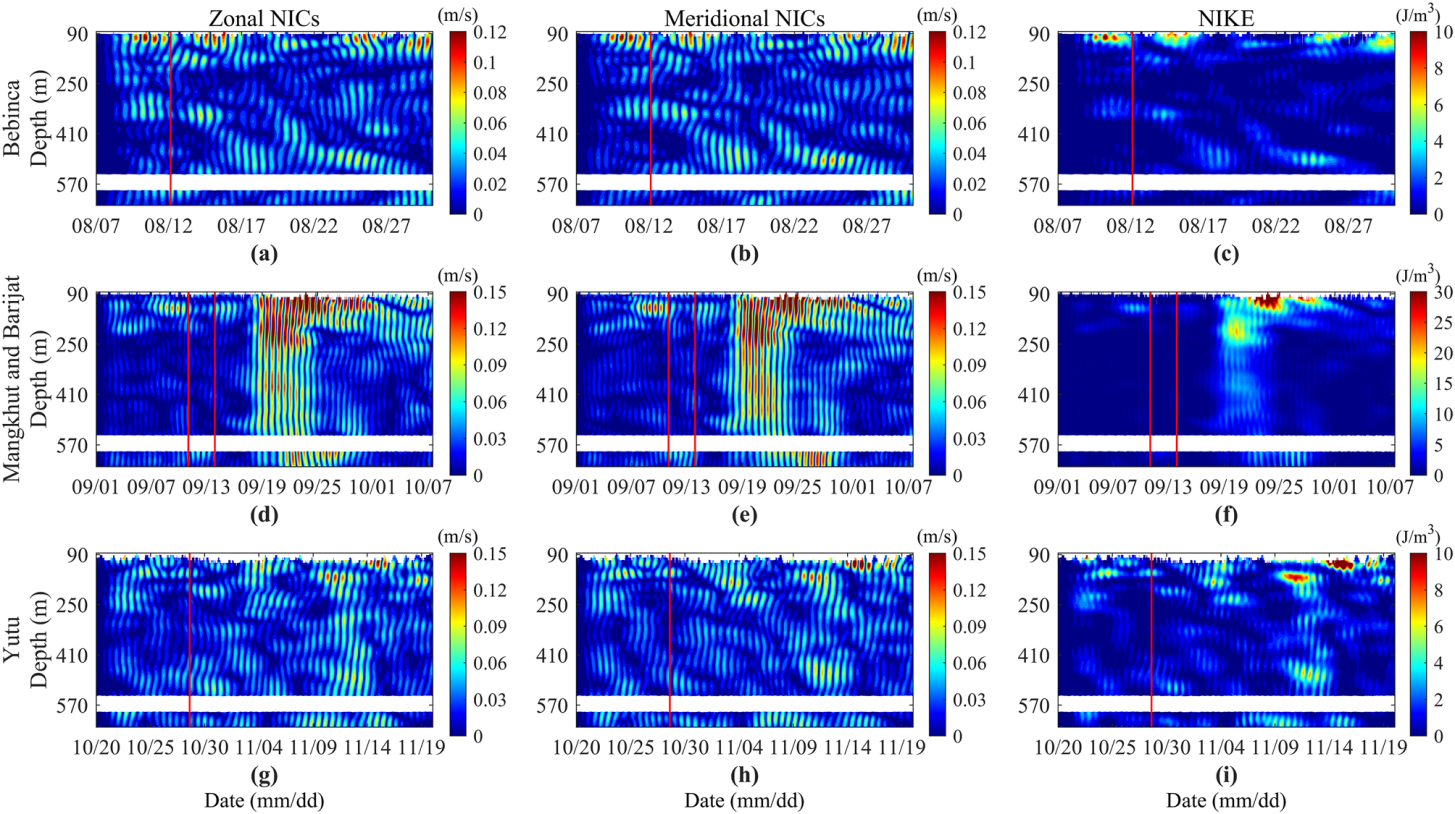
Spatio-temporal distribution of zonal and meridional NICs and NIKE during the passage of typhoons Bebinca (**a–c**), Barijat and Mangkhut (**d–f**), and Yutu (**g–i**), respectively. The red vertical lines are same as Fig. 1.

The spatio-temporal variations of NICs and NIKE induced by typhoons are shown in Fig. 4. After the passage of typhoons Bebinca and Yutu, the NIWs excited by typhoons are in a shallow depth and gradually propagate downward with time. Three days after typhoon Bebinca begin to affect the mooring, the excited NICs reach the maximum of 0.14 m/s at a water depth of 92 m, corresponding to the strongest NIKE of 10.01 J/m^3^. Six days after typhoon Yutu begin to affect the mooring, the excited NICs reach the maximum of 0.13 m/s at a water depth of 109 m, and the strongest NIKE is 9.08 J/m^3^. It should be noted that there are also strong near inertial events in the subsurface between November 9 and November 17. However, this event cannot be explained by the typhoon passing, and understanding of its mechanism merits future study. After the passage of typhoons Mangkhut and Barijat, the excited NIWs are very strong and quickly propagate to the seafloor. The maximum NICs is 0.37 m/s, which is located at the water depth of 84 m on the 10th day after the typhoon passed, and the strongest NIKE is 69.84 J/m^3^.

The NICs and NIKE induced by typhoon Bebinca are the weakest, which are related to the smallest wind speed of 12.35 m/s and the weakest wind stress curl of 0.23 × 10^−5^ N/m^3^ of typhoon Bebinca at the mooring position (Fig. 1b,c). It is worth noting that the maximum wind speeds of typhoon Mangkhut and typhoon Yutu at the mooring are 26.03 m/s and 25.53 m/s respectively (Fig. 1b,c), which are not much different, but the induced NICs and NIKE are very different, indicating that the wind speed is not the decisive factor to directly induce the NIWs. According to the wind stress curl, the maximum wind stress curl of typhoon Mangkhut is 3.99 × 10^−5^ N/m^3^ at the mooring, and typhoon Yutu is 1.75 × 10^−5^ N/m^3^. The former is 2.3 times larger than the latter, and the maximum NICs and NIKE induced by the former are 1.85 and 3.44 times larger than the latter, respectively. Moreover, from the perspective of NICs, in Fig. 4d,e, about 3 days after typhoon Mangkhut begin to affect the mooring, the NICs exceed the depth of 500 m and propagate to the seafloor in the next few days, and the NICs induced by typhoon Yutu do not reach the depth in Fig. 4g,h.

## Discussion

### Depth-averaged NIKE

**Figure 5:**
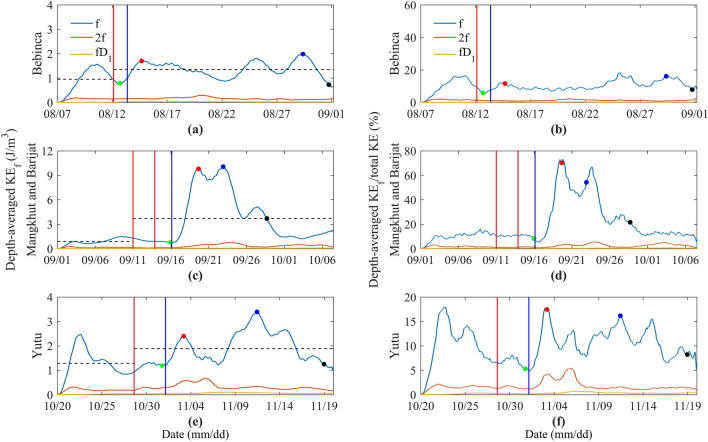
The temporal variations of the depth-averaged NIKE, 2f and fD_1_ energy of band and percentage of total KE during the passage of typhoons Bebinca (**a,b**), Barijat and Mangkhut (**c,d**), and Yutu (**e,f**), respectively. The green dots are the moments when the NIKE started to increase, the red dots are NIKE peaks in the forcing stage, the blue dots are NIKE peaks in the relaxation stage, and the black dots represent the e-folding time of depth-averaged NIKE. The red vertical lines are same as Fig. 1. The blue vertical lines are time of the max wind speed.

Due to the conversion between kinetic energy (KE) and potential energy, NIKE shows the fluctuation in inertial period. In order to better reflect the variation of NIKE, the NIKE is period-smoothed at each layer^5^, then NIKE is averaged in observed depth. Similarly, the energy of 2 f and *f* D_1_ bands are depth-averaged in observed depth (Fig. 5a,c,e). For presenting the temporal variations of the KE of different frequency (KE_*f*_), the proportion of NIKE, 2 f, *f* D_1_ in total KE are calculated (Fig. 5b,d,f).

During the passage of the typhoons, the depth-averaged NIKE showes two distinct peaks in moments near the maximum of wind speed at mooring and after the passage of typhoons, corresponding to the forcing and relaxation stages (Fig. 5). As shown in Fig. 5a, before the passage of typhoon Bebinca, the time-averaged NIKE at mooring is about 0.95 J/m^3^. After the typhoon passed, the time-averaged NIKE increases to 1.34 J/m^3^, and the maximum NIKE is 1.98 J/m^3^. Typhoon Bebinca is far away from the mooring position, the intensity of the typhoon is weak, and the wind stress curl is small, so the change of the near-inertial oscillation induced by typhoon is weak. During the passage of the typhoon, it takes 2.0 days from the moment when the NIKE starts to increase to the first peak, the interval between the first NIKE peak and the second peak is 14.7 days, and then it takes 2.4 days to reach the e-folding time. The typhoon takes about 19.1 days from the time it starts forcing the ocean to the time its impact on the ocean disappears. Before the passage of typhoons Mangkhut and Barijat (Fig. 5b), the time-averaged NIKE at mooring is about 0.87 J/m^3^. After the typhoon passed, the time-averaged NIKE increases to 3.72 J/m^3^, and the maximum NIKE is 10.07 J/m^3^. Both typhoons passe over the mooring. Especially typhoon Mangkhut has a large wind stress curl, which has a great effect on the near-inertial oscillation. During the passage of the typhoon, it takes 3.7 days from the moment when the NIKE start to increase to the first peak, the interval between the first NIKE peak and the second peak is 3.3 days, and then it takes 5.8 days to reach the e-folding time. The typhoon takes about 12.8 days from the time it starts forcing the ocean to the time its impact on the ocean disappears. Before the passage of typhoon Yutu (Fig. 5c), the time-averaged NIKE at mooring is about 1.28 J/m^3^. After the typhoon passed, the time-averaged NIKE increases to 1.89 J/m^3^, and the maximum NIKE is 3.39 J/m^3^. Typhoon Yutu also passes over the mooring, but it has attenuated to a very weak intensity when it passes the mooring. Its wind stress curl is small, so the near-inertial oscillation is relatively weak. During the passage of the typhoon, it takes 2.4 days from the moment when the NIKE starts to increase to the first peak, the interval between the first NIKE peak and the second peak is 8.2 days, and then it takes 7.5 days to reach the e-folding time. The typhoon takes about 18.1 days from the time it starts forcing the ocean to the time its impact on the ocean disappears.

Figure 5b,d,f shows similar results. After the passage of typhoons, the proportion of NIKE to the total KE in the forcing stage begins to increase to a peak, the proportion of NIKE in the relaxation stage begins to increase to the second peak, and then it starts to decrease, which is basically consistent with the change trend of NIKE, except that there is a slight deviation in the peak in the relaxation stage after the typhoons Mangkhut and Barijat passes over the mooring, while the peaks of the forcing stage and relaxation stage of other typhoons are exactly the same. Table 2 shows the average proportion of the KE_*f*_ to the total KE before and after the passage of each typhoon. It can be seen that after each typhoon passed, except for the NIKE, the KE in the *f* D_1_ frequency band also increases, which is caused by the nonlinear interaction between NIWs and diurnal internal tides^55^.

**Table 2:** Average proportion of the depth-averaged NIKE, 2 f and *f* D_1_ energy of band in total KE during the passage of typhoons.

Typhoon	Bebinca		Mangkhut and Barijat		Yutu	
Frequency bands	Before the passage	After the passage	Before the passage	After the passage	Before the passage	After the passage
*f*	9.93	10.46	9.89	23.90	10.22	10.27
*2 f*	1.46	1.24	1.61	2.38	1.51	1.88
*f* D_1_	0.07	0.21	0.19	0.32	0.16	0.33

**Figure 6:**
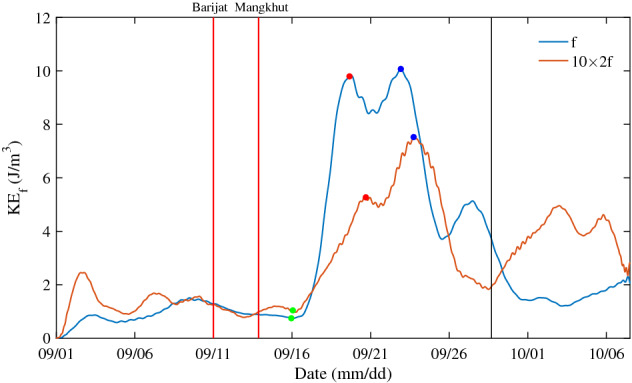
Depth-averaged NIKE and 10 times the KE in 2 *f* energy of band during the passage of typhoons Mangkhut and Barijat. The red vertical lines are same as Fig. 1. The black vertical line denotes the e-folding time of depth-averaged NIKE.

It can be seen that, after the passage of typhoons Mangkhut and Barijat, the power spectrum has obvious peaks near 2 *f* in Fig. 3e, g, the current velocity with a frequency of 2 *f* is obtained by bandpass filtering, and its KE is calculated and compared to NIKE (Fig. 6). After the passage of the typhoons, the time difference between NIKE and 2 *f* band KE beginning to increase is 0.09 days, the time difference between the first peaks of the two KEs is 1 day, and the time difference between the second peaks is 0.82 days. The correlation coefficient between NIKE and 2 *f* band KE is 0.35 before the passage of typhoon, and the correlation coefficient reaches 0.83 after the passage of the typhoons, showing a good consistency. This indicates that the fluctuation of 2 *f* band is generated at the mooring position and enters the sea, which may be related to the impact of quasi-geostrophic flow on NIWs^56^.

**Figure 7:**
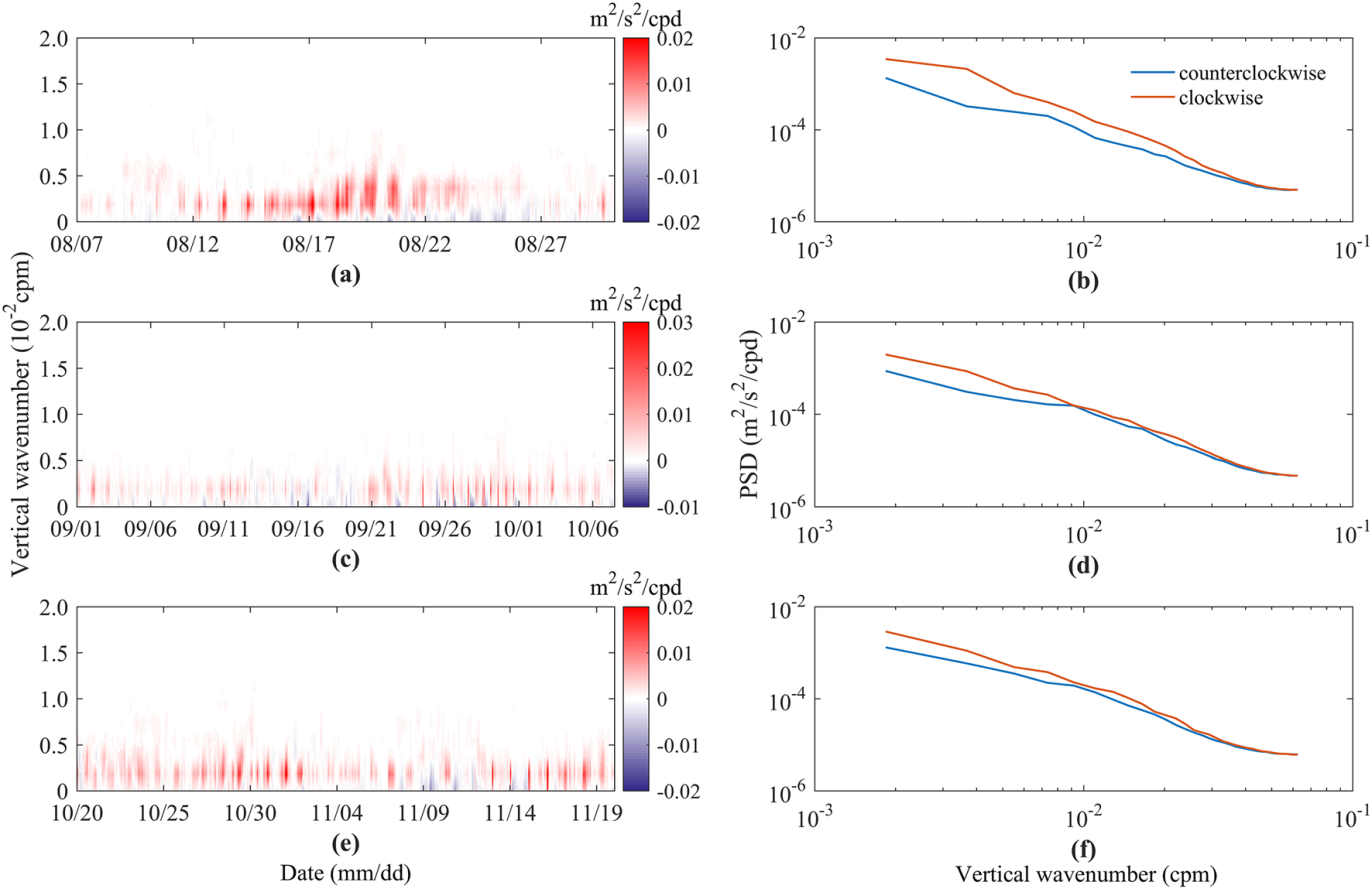
Rotary vertical wavenumber difference between clockwise and counterclockwise components and time-averaged rotary vertical wavenumber spectra during the passage of typhoons (**a,b**) Bebinca, (**c,d**) Barijat and Mangkhut, and (**e,f**) Yutu. The blue and orange solid lines represent the time-averaged counterclockwise and clockwise rotary components, respectively. cpm stands for “cycles per meter”.

To further study the propagation characteristics of NIKE, we use the rotary vertical wavenumber spectra of the NICs. Figure 7a,c,e illustrates the temporal evolution of the difference between the clockwise rotary spectra and the counterclockwise rotary spectra. A positive (negative) value indicates that the energy propagates downward (upward). Note that the color scale of the colormap is asymmetrical. Figure 7b,d,f shows the time-averaged rotary vertical wavenumber spectra of the NICs after the passage of typhoons. The results are similar, and NIKE mainly propagates downward, with the smaller wavenumber band (< 0.005 cpm) being the most significant. Moreover, the phenomenon of the upward propagation of energy is present, which is consistent with the conclusion of Gill^11^, that is, after the passage of typhoon, the upward and downward propagations of energy in the ocean exist at the same time. This study shows that the upward propagating energy mainly occurs in the relaxation stage. The time-averaged rotary vertical wavenumber spectra show that the clockwise component is always larger than the counterclockwise component, especially in the wavenumber band less than 0.01 cpm.

### Temperature response of the ocean

**Figure 8:**
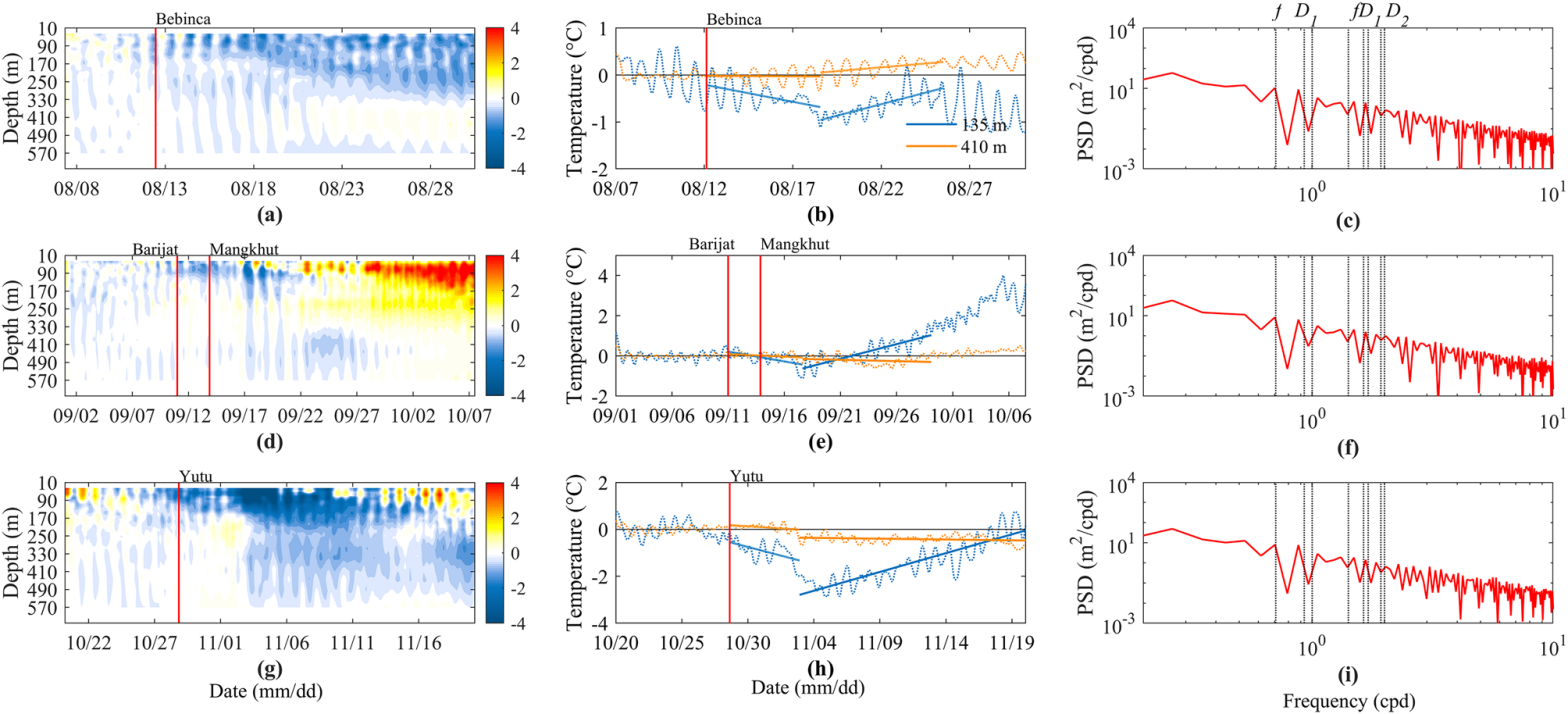
Spatio-temporal distribution of temperature anomaly (temperature minus the average temperature of the 4 days before the passage of typhoons, (**a,d,g**)), time series of temperature at a depth of 135 m and 410 m (**b,e,h**), and spectra of average vertical displacement of the isotherms from 8 °C to 18 °C (**c,f,i**) during the passage of typhoons Bebinca (**a–c**), Barijat and Mangkhut (**d–f**), and Yutu (**g–i**), respectively. The red vertical lines are same as Fig. 1. The black lines are thermocline intensity. The blue lines are temperature at the depth of 135 m. The orange lines are temperature fitting curves. The eight black vertical lines are same as Fig. 3.

In order to analyze the effect of typhoon on subsurface seawater temperature, after 25 h of low-pass filtering, we take the time average of the temperature of the four days before the passage of typhoons as the background temperature, and obtain the spatio-temporal distribution of temperature anomalies by subtracting this background temperature from the observed temperature during the passage of typhoons (Fig. 8a,d,g), and give the temperature anomaly sequences at the depth of 135 m, 250 m and 410 m, respectively (Fig. 8b,e,h). As mentioned earlier, the process of typhoons impact on the thermocline is also divided into two stages: forcing and relaxation stages. It can be seen that in the forcing stage after the passage of typhoons, there are alternately large cold and warm anomalies, mainly showing that the upper layer is cooling and the lower layer is warming. In the forcing stage, the maximum cold anomalies are about 0.47 °C, 0.64 °C and 0.73 °C at the water depth of 135 m during the passage of typhoons Bebinca, Barijat and Mangkhut, and Yutu, respectively. In the relaxation stage, the upper layer temperature increases, while the lower layer temperature decreases. Spectra of average vertical displacement of the isotherms from 8 °C to 18 °C are dominated by local inertial frequency and diurnal internal tides (Fig. 8c,f,i), indicating that typhoons will affect the vertical distribution of temperature, and near-inertial oscillation has become one of the factors controlling temperature changes.

**Figure 9:**
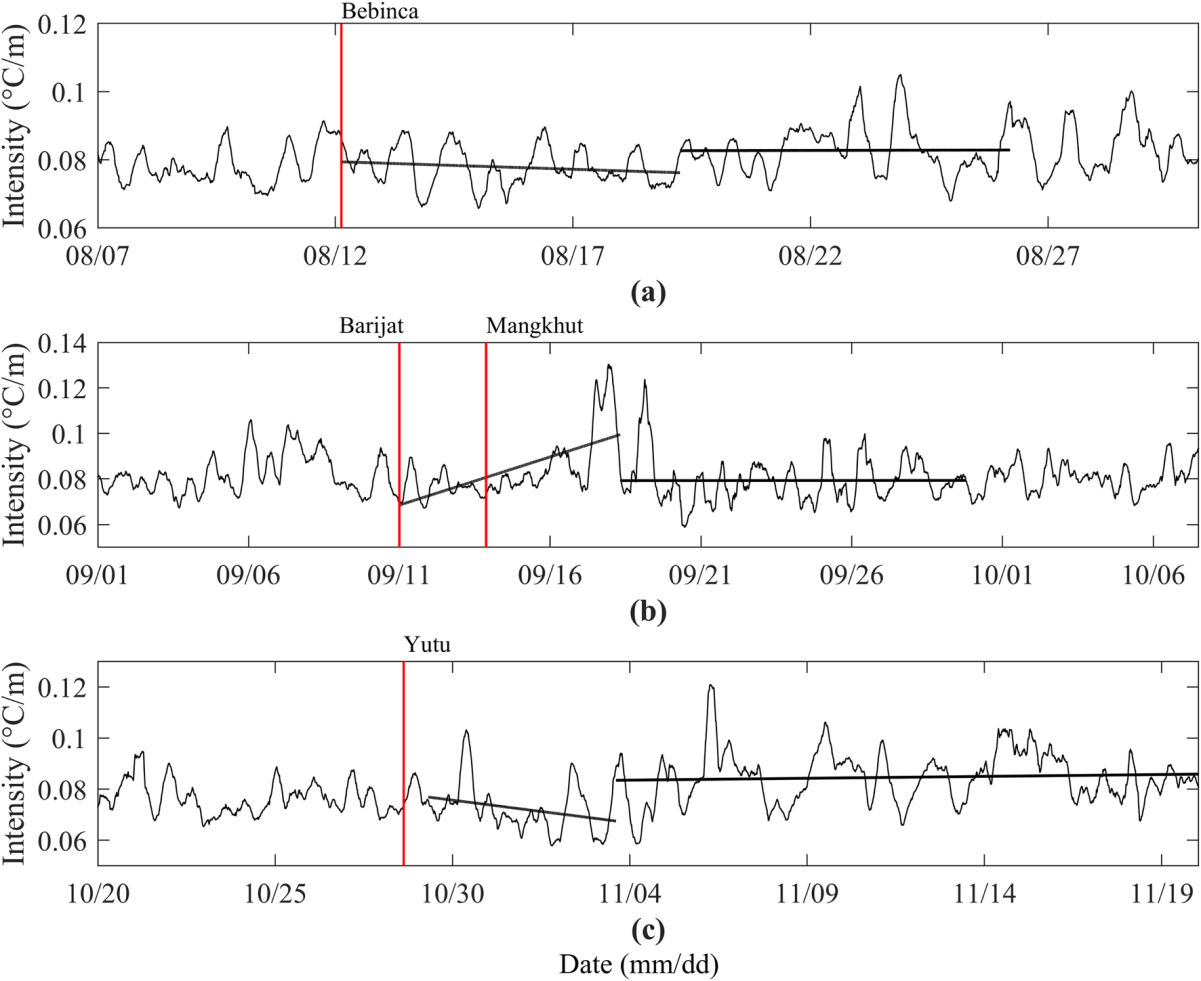
Time series of intensity of the thermocline during the passage of typhoons Bebinca (**a**), Barijat and Mangkhut (**b**), and Yutu (**c**), respectively. The red vertical lines are same as Fig. 1. The black straight lines are fitting curves of intensity of the thermocline.

Combining the characteristics of the changes in the depth of the thermocline (Fig. 2) and studying the intensity of the thermocline (Fig. 9), we can see that in the forcing stage, typhoons Bebinca and Yutu cause the rise of the thermocline, the thickness of the thermocline decreases, and the intensity of the thermocline also decreases, indicating that the temperature difference between the upper and lower boundaries of the thermocline decrease, which is consistent with the above results that the upper ocean temperature decreases and the lower ocean temperature increases. In the case of typhoons Barijat and Mangkhut, the thermocline also rises, and the thickness of the thermocline decreases, but the intensity of the thermocline increases, which only shows that the temperature change does not cancel out the effect of the thickness change on the intensity. It is impossible to see how the temperature changes from here alone. From the change of thermocline, there is a front or an edge of eddy crossing the mooring, which may be the reason for the enhancement of thermocline intensity and the rapid downward propagation of NIWs. In the relaxation stage, the depth of the thermocline becomes deeper, the thickness increases, and the intensity of the thermocline increases, indicating that the temperature difference between the upper and lower boundaries of the thermocline increases, so the upper ocean temperature warms up and the lower ocean temperature decreases. Therefore, after the passage of typhoons, the temperature shows obvious stratification vertical structure. In the forcing stage, the upper layer becomes colder, the lower layer becomes warmer, and the thickness and intensity of the thermocline decrease. In the relaxation stage, the upper layer warms and the lower layer cools, and the thickness and intensity of thermocline increase.

### Current structure of NIWs

**Figure 10:**
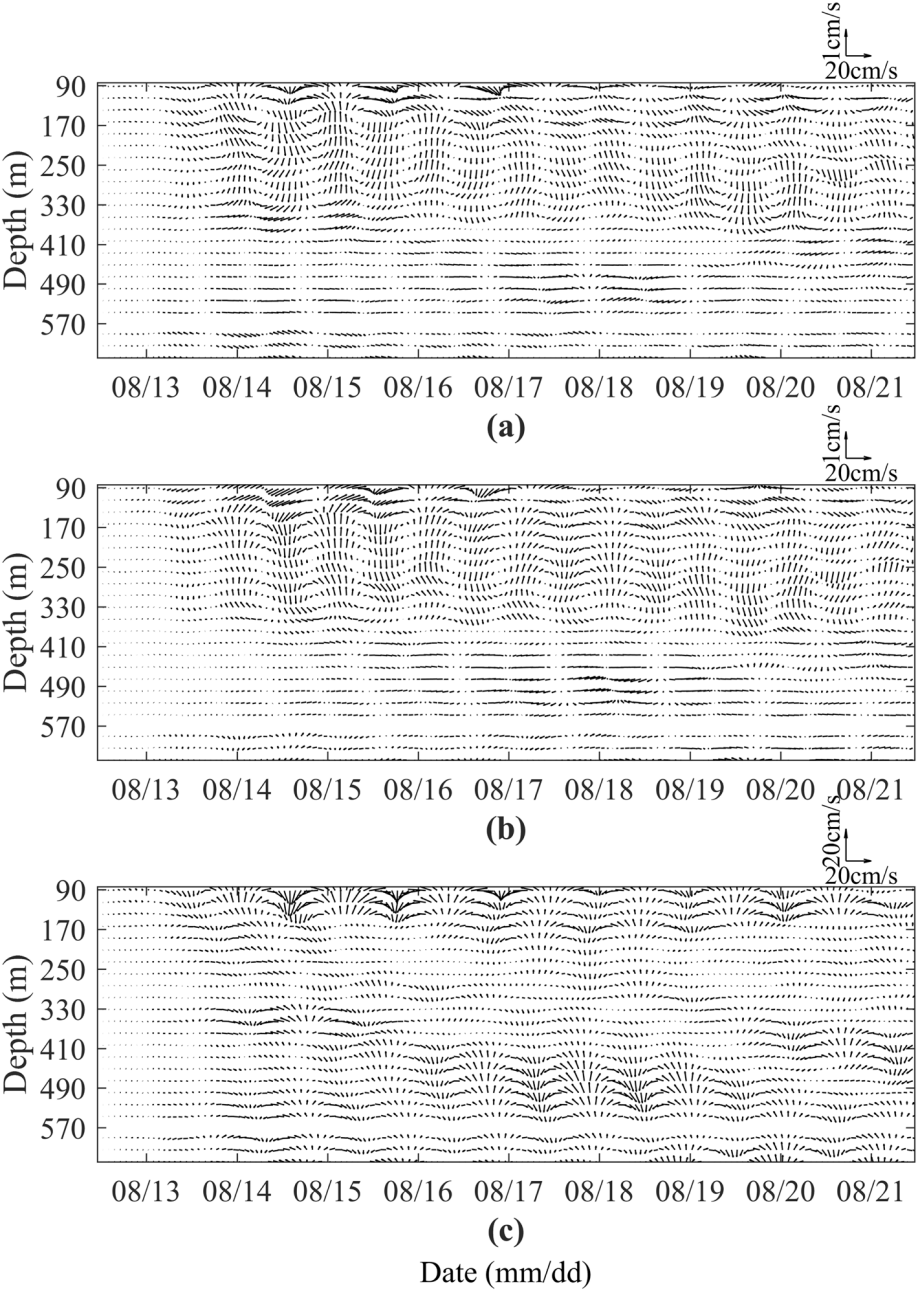
Spatio-temporal distribution of the near-inertial zonal-vertical current velocity (**a**), meridional-vertical current velocity (**b**) and zonal-meridional current velocity (**c**) during the passage of typhoons Bebinca.

**Figure 11:**
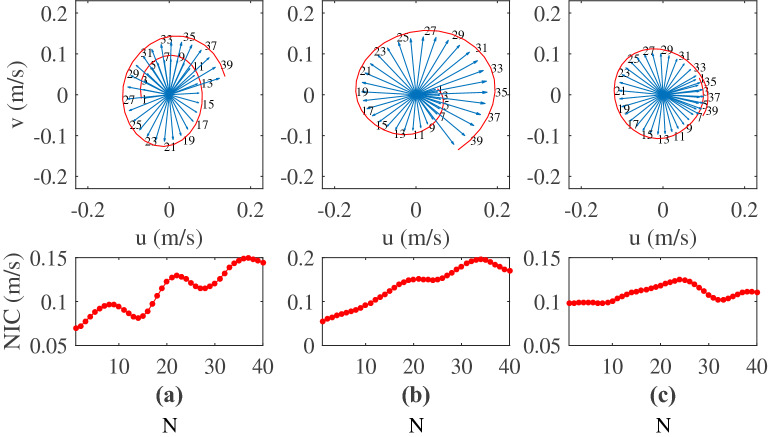
Variations of the NICs with time after the passage of typhoons Bebinca (**a**), Barijat and Mangkhut (**b**), and Yutu (**c**), respectively. The red line is the NICs, “N” is the serial number of the selected NICs, and the time difference between the two adjacent serial numbers is 1 h.

In order to show the current structure of the NIWs, we take the typhoon Bebinca as an example to give a spatio-temporal distribution of the near-inertial horizontal current velocity and vertical current velocity in Fig. 10. It is worth noting that the vertical motion of ADCP affects the observed vertical current velocity, so here we use the measured vertical current velocity and the vertical motion velocity of ADCP to synthesize the true vertical current velocity. The results show that in the vertical direction (Fig. 10a,b), there are two antisymmetric rotary vortices in a near-inertial period, and the structure is similar to the Langmuir circulation. Generally, the horizontal NICs is much larger than the vertical velocity, and the stronger the NIWs is, the larger the horizontal NICs is than the vertical NICs. In the horizontal direction, the NICs rotates clockwise with time (Fig. 10c). The period of NIWs induced by typhoon Bebinca is about 27 h (Fig. 10c), the period induced by typhoon Barijat and Mangkhut is about 33 h (Figure omitted), and the period induced by typhoon Yutu is about 33 h (Figure omitted). In order to more intuitively show the change of current vectors, Fig. 11 shows the change of the near-inertial current vectors with time after the passage of typhoons. The periods of the NIWs induced by typhoon Bebinca are between 27 and 30 h, the periods induced by typhoon Barijat and Mangkhut are about 33–37 h, and the periods induced by typhoon Yutu are about 31–35 h.

## Summary

The strong atmospheric forcing of typhoons can induce NICs in the upper ocean. From 21 July to 20 December 2018, a mooring is deployed in the northern of the SCS. The ADCPs are located at a water depth of 580 m, and sent signals upward-looking and downward-looking to measure the current velocity from 90 to 650 m. CTDs and temperature loggers measure temperature and depth from 50 to 580 m. The purpose is to study the thermal and dynamic effects of typhoons Bebinca, Barijat, Mangkhut and Yutu on the ocean through the in situ mooring observations of temperature and current velocity, and to analyze the differences and effects of NIWs generated by different typhoons. These data support the following conclusions.

During the observation period, the passage of typhoons increase the current velocity in the subsurface layer, mainly due to the enhancement of the NICs. Through the power spectrum analysis, except for the weak NICs induced by the typhoon Bebinca, the NICs induced by other typhoons are relatively obvious, even exceeding the spectral energy of the diurnal internal tides. After the passage of typhoons, the NICs increase, corresponding to the increase of NIKE, and the maximum NIKE is confined to depths above 150 m.

After the passage of typhoon Bebinca, it takes 2 days to reach the forcing peak, and then the whole relaxation stage is the adjustment of geostrophic balance, which lasts for 17.1 days. after the passage of typhoons Barijat and Mangkhut, it takes 3.7 days to reach the forcing peak, and the geostrophic balance adjustment lasts 9.1 days. After the passage of typhoon Yutu, it takes 2.4 days to reach the forcing peak, and the geostrophic balance adjustment lasts for 15.7 days. Combined with the wind forcing intensity, we draw a conclusion that for the stronger (weaker) wind forcing, the longer (shorter) the response time of the ocean to the atmospheric forcing is, the stronger (weaker) external force the ocean might be subjected to, and the shorter (longer) the required time for geostrophic balance adjustment is.

Through the analysis of the rotary spectrum, we know that both the tidal component and the near-inertial component are dominated by the clockwise component, which indicates that the energy mainly propagates downward. The downward propagation mainly occurs in smaller wavenumber band (< 0.01 cpm), and there also has upward propagating energy in the ocean after the passage of typhoon, which mainly occurs in the relaxation stage.

After the passage of typhoons, in addition to the increase in NIKE, the KE in the *f* D_1_ frequency band also increases, which indicates that the NIWs have a nonlinear interaction with the diurnal internal tides^50^. Moreover, after the passage of typhoons Barijat and Mangkhut, the KE in 2 *f* frequency band increases significantly, and has the similar trend to the NIKE, which indicates that the fluctuation of 2 *f* band is generated at the mooring position and entered the sea.

Typhoons affect the vertical distribution of temperature. After the passage of typhoons, the temperature shows obvious stratification vertical structure. In the forcing stage, the upper layer becomes colder, the lower layer becomes warmer, and the thickness and intensity of the thermocline decrease. In the relaxation stage, the upper layer warms and the lower layer cools, and the thickness and intensity of thermocline increase.

The current structure of the NIWs shows that in the vertical direction, there are two antisymmetric rotary vortices in a near-inertial period, and the structure is similar to the Langmuir circulation. Generally, horizontal NICs is much larger than the vertical velocity, and the stronger the NIWs is, the larger the horizontal NICs is than the vertical NICs. In the horizontal direction, the NICs rotate clockwise with time, and the NIWs induced by different typhoons have main frequency bands near the inertial frequency.

## Data Availability

Hourly 10 m wind speed data obtained through the European Center for Medium-range Weather Forecasts Website (https://www.ecmwf.int/en/forecasts/datasets/browse-reanalysis-datasets, accessed on 21 March 2021). The typhoon best track dataset obtained through the China Typhoon Network (tcdata.typhoon.org.cn, accessed on 21 March 2021).
